# Concurrent Multiscale Modelling of Thermomechanical Responses of Heterogeneous Partition Walls

**DOI:** 10.3390/ma18204744

**Published:** 2025-10-16

**Authors:** Shige Wang, Sen Yang, Yang Li, Lian Huang, Yanming Xu, Heng Zhang, Pei Li

**Affiliations:** 1 College of Architecture and Civil Engineering, Xinyang Normal University, Xinyang 464000, China; wangshige1979@163.com (S.W.); yangsen7887@163.com (S.Y.); 2 Henan International Joint Laboratory of Structural Mechanics and Computational Simulation, College of Architecture and Civil Engineering, Huanghuai University, Zhumadian 463000, China; xuyanming@ustc.edu; 3 Intelligent Power System Co., Ltd., Wuhan 430056, China; xxliyang@foxmail.com; 4 Solux College of Architecture and Design, University of South China, West Changsheng Road, Hengyang 421001, China; 18384995481@163.com; 5 International Machinery Center, Department of Mechanical Engineering, Xi’an Jiaotong University, Xi’an 710049, China; 6 Department of Mechanical Engineering, National University of Singapore, 21 Lower Kent Ridge Road, Singapore 119077, Singapore; 7 Centre for Industrial Mechanics, Institute of Mechanical and Electrical Engineering, University of Southern Denmark, 6400 Sønderborg, Denmark

**Keywords:** multiscale finite element method, direct FE^2^ method, thermo-mechanical coupling, partition wallboard, representative volume element, multipoint constraints

## Abstract

Partition walls are widely used in engineering structures, and their thermomechanical performance has a significant influence on overall safety and durability. Under extreme conditions, such as high temperatures, these walls are subjected to complex thermal expansion, stress development, and deformation, which may compromise structural stability. Analyzing full-field deformation of parathion walls with high accuracy is a burden for classical fine-scale finite element methods. To address these challenges, this study applies a multiscale finite element method to investigate the coupled thermomechanical behavior of partition walls, providing a more computationally efficient alternative to conventional single-scale models. The method effectively captures thermal–mechanical interactions in walls composed of solid steel, porous steel, and composite plates. Numerical simulations confirm the accuracy and efficiency of the proposed approach, demonstrating its suitability for practical engineering applications. The results offer a reliable basis for optimizing partition wall design, improving energy performance, and ensuring structural integrity under demanding operating conditions.

## 1. Introduction

Partition walls are essential components in modern architecture and engineering. Their safety, durability, and stability are critical design considerations. Under complex service conditions, particularly high temperatures, partition walls can experience thermal expansion, stress accumulation, and deformation. These effects may degrade structural performance or even cause catastrophic failure [1]. Thermo-mechanical coupling analysis provides a vital tool to evaluate partition wall behavior under extreme conditions. This analysis informs robust design, material selection, construction practices, and maintenance protocols.

Current research on temperature distribution and thermal stress in partition walls employs three primary methodologies: numerical simulations, experimental testing, and theoretical analysis. Numerical approaches, such as the boundary element method (BEM) [2,3,4], the finite element method (FEM) [5,6,7], the finite difference method (FDM), and the finite volume method (FVM) [8], are widely utilized for solving heat conduction equations. Experimental techniques, including thermocouples, infrared thermography [9], strain gauges, and digital image correlation (DIC) [10], enable empirical validation and visualization of thermal responses. Theoretical methods, grounded in Fourier’s law of heat conduction and thermoelasticity theory [11,12,13], derive closed-form or semi-analytical solutions under idealized conditions. These analytical approaches remain indispensable for preliminary assessments, numerical model validation, and parametric studies.

Large-scale fire and heating tests on solid steel plates have established benchmark temperature–displacement relationships and degradation patterns under elevated temperatures [14,15]. Experimental investigations on perforated or holed steel plates have confirmed that void geometry significantly alters heat transfer, stiffness, and stress concentration, thereby affecting global deformation modes [16,17]. Likewise, numerous tests on fiber-reinforced composite plates have demonstrated pronounced temperature-dependent reductions in stiffness and interfacial integrity, as well as distinctive failure modes under combined thermal and mechanical loading [18,19]. Collectively, these experimental findings provide essential physical evidence that underpins the boundary conditions, material degradation laws, and structural responses adopted in the present Direct FE^2^ simulations, ensuring that the numerical results are firmly grounded in engineering practice.

Despite these advancements, critical limitations remain. Numerical simulations, although accurate, require high computational costs, especially for multilayered or heterogeneous materials, limiting their application in real-time or large-scale projects [20]. Experimental methods often face issues with repeatability and generalizability due to sensor precision and environmental control constraints [21]. Most theoretical models assume steady-state or quasi-steady-state behavior, failing to capture transient phenomena and nonlinear heat conduction [22]. Modern partition walls, designed with multiscale features such as porous or composite structures, further challenge traditional analysis methods due to the high computational demands.

A promising solution to address this challenge may be the emerging concurrent multiscale modeling methodologies, and a commonly used one is the finite element squared (FE^2^) method [23] originally proposed by Feyel [24]. Rooted in the Hill–Mandel homogenization principle [25,26], FE^2^ transfers macroscopic strain fields to microscale representative volume elements (RVEs) and retrieves homogenized stress responses, enabling high-fidelity simulations of heterogeneous materials [27]. Conventional FE^2^ implementations, however, require iterative cross-scale communication at each Gauss integration point, leading to prohibitive computational overhead and implementation challenges. Tan et al. [28] introduced the Direct FE^2^ to overcome these limitations. By integrating macro- and microscale models into a unified framework via multi-point constraints (MPCs), and equivalence of internal virtual work, this approach achieves efficient and stable coupling between RVEs and macroscopic finite element models [29]. The Direct FE^2^ method has demonstrated success across diverse applications, including static and dynamic mechanical analysis [30,31,32], multiple physical problems [33,34,35,36], optimization problems [37,38,39,40]. Especially, Zhi [33,35] extended the Direct FE^2^ method toward the thermo-mechanical problems, and accurately predicted the cure-induced deformation in composite laminates, exhibiting the enormous potential of this method in addressing engineering problems.

However, it seems that multiscale modeling methodologies have not yet been applied to the plentiful heterogeneous materials and structures in civil engineering, such as the multiscale partition walls widely used in modern buildings. This work is directed to explore the possibility of using the Direct FE^2^ method to simulate the complicated thermomechanical responses of multiscale partition walls in the real world.

## 2. Theory of Concurrent Multiscale Modeling

Since partition walls in actual applications generally bear time-dependent temperature and static loadings, the concurrent multiscale modeling theory in this work is derived from the governing equations for coupled thermomechanical problems considering both static and transient heat conductions. It is detailed as below:

### 2.1. Governing Equations for Coupled Thermomechanical Problems

The governing equations of thermal and mechanical fields in transient thermomechanical problems can be generally written as

(1) $$\rho \dot{U}=-div\;q_{i}+\rho r\;\rho {\ddot{u}}_{i}=div\sigma_{ij}+\rho b_{i}$$

where ρ and $\dot{U}=C_{p}\frac{dT}{dt}$ are the material’s density and the rate of the material’s internal energy, respectively, and $C_{p}$ represents the material’s specific heat. $q_{i}={-k}_{ij}T_{,j}$ is the heat flux where $k_{ij}$ is thermal conductivity and $T_{,j}$ represents the temperature gradient. r denotes the internal heat source per unit volume, which is set to zero in this study due to the absence of internal heat generation. ${\ddot{u}}_{i}$ denotes the acceleration field, $\sigma_{ij}$ represents Cauchy stress tensor and $b_{i}$ is body force.

### 2.2. Concurrent Multiscale Modeling

This work adopts the Direct FE^2^ method [28,31] for concurrent multiscale modeling of the coupled thermomechanical problems of partition walls. Hence, the FE governing equations need to be reviewed first. For instance, the thermal governing equation (Equation (1)) can be converted into the following weak form for finite element simulations:

(2) $$\int_{V}\;\rho \dot{U}\delta TdV+\int_{V}\;q_{i}\delta T_{,j}dV=\int_{\Gamma }\;q_{\Gamma }\delta Td\Gamma$$

where $\rho \dot{U}\delta T\;$ describes the heat absorbed or released by unit mass of material when temperature changes, also known as the heat capacity term. This term must be considered for transient thermal analysis but vanishes for steady-state problems.

Since partition walls usually bear static mechanical loads, the weak form of the mechanical governing equation can be expressed as follows, whereby the inertia term and body force are ignored:

(3) $$\int_{V}\sigma_{ij}\delta \varepsilon_{ij}dV=\int_{V}\left(\sigma_{ij}^{M}+\sigma_{ij}^{T}\right)\delta \varepsilon_{ij}dV=\int_{\Gamma }t_{i}\delta u_{i}d\Gamma$$

where $\sigma_{ij}=\sigma_{ij}^{M}+\sigma_{ij}^{T}$ and $\sigma_{ij}^{M}=C_{ijkl}\varepsilon_{kl}$, $\sigma_{ij}^{T}=-C_{ijkl}\beta_{kl}\left(T-T_{0}\right)$. $\sigma_{ij}^{M}$ and $\sigma_{ij}^{T}$ represent the stresses caused by mechanical strain $\varepsilon_{kl}$ and thermal expansion due to temperature change $\left(T-T_{0}\right)$, respectively. $C_{ijkl}$ denotes the fourth-order elastic stiffness tensor, $\beta_{kl}$ is the thermal expansion coefficient, T and $T_{0}$ are current and reference temperatures, respectively. u denotes displacement field and the strain tensor is given by $\varepsilon_{ij}=\frac{1}{2}\left(u_{i,j}+u_{j,i}\right)$. $t_{i}$ represents the traction force on the boundary. i,j=1, 2 for 2D problems considered in this work.

For coupled thermomechanical problems, one needs to consider both Equations (2) and (3), and the total internal energy density can be expressed as the summation of the left-hand side of these two equations:

(4) $$\delta H_{den}=\sigma_{ij}\delta \varepsilon_{ij}+\rho \dot{U}\delta T+q_{i}\delta T_{,i}$$

**Figure 1:**
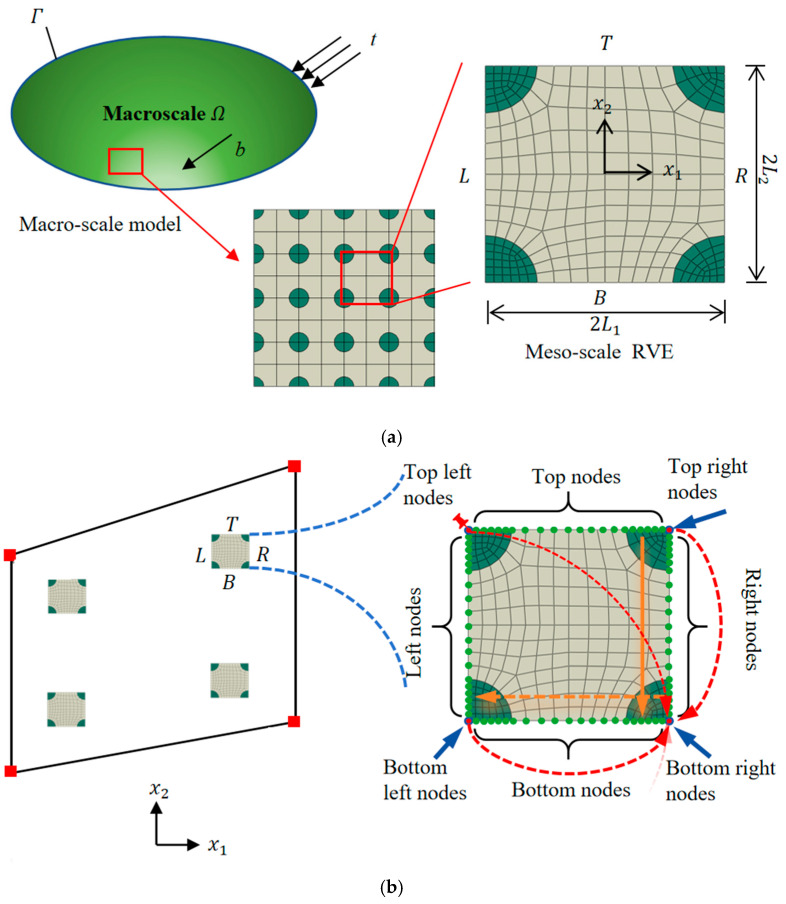
(**a**) Illustration of a multiscale material and its meso-scale RVE, and (**b**) its schematic diagram of a Direct FE^2^ model and the corresponding direct numerical simulation (DNS).

It shall be noted that the macro-scale responses of multiscale partition walls are dominated by micro/meso-scale patterns. To simultaneously simulate the macro- and meso-scale responses, the Direct FE^2^ method models the macro-structure using coarse macro-meshes and establishes a representative volume element (RVE) to represent the meso-scale details at each Gaussian point of every macro-element. Figure 1 illustrates an example of fiber reinforced composite panel modeled using the Direct FE^2^ method. The RVE, as shown in Figure 1a, has a length and width of $2l_{1}\;\mathrm{and}\;2l_{2}$, respectively. Variables denoted as $\hat{◼}$ are assessed at meso-scale, otherwise at macro-scale. Similarly, the gradient of a mesoscale variable is expressed as ${\hat{◼}}_{,i}=\frac{\partial \hat{◼}}{\partial {\hat{x}}_{i}}(i=1,2)$.

Thus, the average volume of total internal energy evaluated at mesoscale is

(5) $$\langle \delta \hat{H}\rangle =\frac{1}{\left|\hat{V}\right|}\int_{\hat{V}}\;\left({\hat{\sigma }}_{ij}\delta {\hat{\varepsilon }}_{ij}+\hat{\rho }\dot{\hat{U}}\delta \hat{T}+{\hat{q}}_{i}\delta {\hat{T}}_{i}\right)d\hat{V}$$

where $\langle *\rangle =\frac{1}{\left|\hat{V}\right|}\int_{\hat{V}}\;*\;d\hat{V}$ denotes volume average and $\left|\hat{V}\right|$ is the volume of the RVE.

### 2.3. Transition from Macro- to Meso-Scale

The macro to mesoscale transition of coupled thermo-mechanical fields depends on the constraints of strain and temperature, whereby the macro-scale strain $\varepsilon_{ij}$ and temperature gradient $T_{,i}$ are recovered by the volume average of meso-scale fields, i.e.,

(6) $$\varepsilon_{ij}=\frac{1}{\left|\hat{V}\right|}\int {\hat{\varepsilon }}_{ij}d\hat{V}\;T_{,i}=\frac{1}{\left|\hat{V}\right|}\int {\hat{T}}_{,i}d\hat{V}$$

For concurrent multiscale modeling assuming the first-order homogenization, the meso-scale field can be decomposed into a smoothly varying field superposed with a rapidly fluctuating field, i.e.,

(7) $${\hat{u}}_{i}=u_{i}+\nabla_{j}u_{i}{\hat{x}}_{j}+{\hat{u}}_{i}^{*}\;\hat{T}=T+\nabla_{i}T{\hat{x}}_{i}+{\hat{T}}^{*}$$

where ${\hat{u}}_{i}^{*}$ and ${\hat{T}}^{*}$ are the meso-scale fluctuating fields for displacement and temperature, respectively, while ∇ denotes the gradient operator. Substitution of Equation (7) into the kinetic constraints in Equation (6) implies that

(8) $$\int {\hat{u}}_{i,j}^{*}d\hat{V}=0\;\int {\hat{T}}_{,i}^{*}d\hat{V}=0$$

These two conditions are automatically satisfied if periodic boundary conditions are prescribed to both the mesoscale displacement and temperature fields in the multiscale modeling. Moreover, periodic boundary conditions prescribed to the mesoscale RVEs also ensure the energy equilibrium between the macro- and mesoscales, which is detailed as follows.

### 2.4. Transition from Meso- to Macro-Scale

Concurrent multiscale modeling requires satisfaction of the Hill–Mandel homogenization condition, i.e., the total internal energy assessed at macro-scale in Equation (4) must equate to the volume average of meso-scale internal energy in Equation (5). Substitution of Equation (7) into Equation (5) enables the volume average of meso-scale internal energy to be rewritten as

(9) $$\langle \delta \hat{H}\rangle =\frac{1}{\left|\hat{V}\right|}\int_{\hat{V}}\;[{\hat{\sigma }}_{ij}{(\nabla }_{j}\delta u_{i}+\delta {\hat{u}}_{i,j}^{*})+\hat{\rho }\dot{\hat{U}}\delta \hat{T}+{\hat{q}}_{i}(\delta T_{,i}+\delta {\hat{T}}_{,i}^{*})]d\hat{V}$$

which can be further re-expressed as follows by using Gauss’s divergence theorem:

(10) $$\langle \delta \hat{H}\rangle =\frac{1}{|\hat{V}|}\int_{\hat{V}}\left({\hat{\sigma }}_{ij}\nabla_{j}\delta u_{i}+\hat{\rho }\dot{\hat{U}}\delta \hat{T}+{\hat{q}}_{i}\delta T_{,i}\right)d\hat{V}+\frac{1}{|\hat{V}|}\int_{\hat{\Gamma }}\left({\hat{t}}_{i}\delta {\hat{u}}_{i}^{*}+{\hat{q}}_{n}\delta {\hat{T}}^{*}\right)d\hat{\Gamma }$$

by noting that

(11) $${\hat{\sigma }}_{ij}{\hat{n}}_{j}={\hat{t}}_{i}\;{\hat{q}}_{i}{\hat{n}}_{i}={\hat{q}}_{n}$$

where ${\hat{n}}_{j}$ denotes the unit outward normal of RVE’s boundary $\hat{\;\Gamma }$, ${\hat{t}}_{i}$ and ${\hat{q}}_{n}$ denote the traction and heat flux acting on the boundary $\hat{\;\Gamma }$.

To enforce the Hill–Mandel homogenization condition, the work and heat related to the meso-scale fluctuation ${\hat{u}}_{i}^{*}$ and ${\hat{T}}^{*}$ in Equation (10) must be vanished. This can be achieved by applying periodic boundary conditions (PBCs):

(12) $$\delta {\hat{u}}_{k}^{*}{|}_{R}=\delta {\hat{u}}_{k}^{*}{|}_{L}\left(k=1,2\right)\;\delta T^{*}{|}_{R}=\delta T^{*}{|}_{L}\;\delta {\hat{u}}_{k}^{*}{|}_{T}=-\delta {\hat{u}}_{k}^{*}{|}_{B}\left(k=1,2\right)\;\delta T^{*}{|}_{T}=-\delta T^{*}{|}_{B}$$

where **R**, **L**, **T** and **B** denote the right, left, top and bottom boundaries of the RVE, respectively. The resulting traction and heat flux on the boundary are anti-periodic:

(13) $${\hat{t}}_{k}{|}_{R}=-{\hat{t}}_{k}{|}_{L}\;{\hat{q}}_{n}{|}_{R}=-{\hat{q}}_{n}{|}_{L}\;{\hat{t}}_{k}{|}_{T}=-{\hat{t}}_{k}{|}_{B}\;{\hat{q}}_{n}{|}_{T}=-{\hat{q}}_{n}{|}_{B}$$

This eliminates the boundary integral term involving meso-scale fluctuation ${\hat{u}}_{i}^{*}$ and ${\hat{T}}^{*}$ in Equation (10). Hence, the average volume of meso-scale internal energy can be rewritten as

(14) $$\langle \delta \hat{H}\rangle =\frac{1}{\left|\hat{V}\right|}\int_{\hat{V}}\;\left({\hat{\sigma }}_{ij}\nabla_{j}\delta u_{i}+\hat{\rho }\dot{\hat{U}}\delta \hat{T}+{\hat{q}}_{i}\delta T_{,i}\right)d\hat{V}$$

The Hill-Mandel homogenization condition requires

(15) $$\langle \delta \hat{H}\rangle =\delta H_{den}$$

Hence, comparison between Equations (4) and (14) yields the macro-scale stress tensor and heat flux:

(16) $$\sigma_{ij}=\frac{1}{\left|\hat{V}\right|}\int_{\hat{V}}\;{\hat{\sigma }}_{ij}d\hat{V}\;q_{i}=\frac{1}{\left|\hat{V}\right|}\int_{\hat{V}}\;{\hat{q}}_{i}d\hat{V}$$

The heat capacity term $\hat{\rho }\dot{\hat{U}}\delta \hat{T}$ in Equation (4) is defined as

(17) $$\rho \dot{U}\delta T=\langle \hat{\rho }\dot{\hat{U}}\delta \hat{T}\rangle =\langle \hat{\rho }\rangle \langle \dot{\hat{U}}\rangle \langle \delta \hat{T}\rangle$$

## 3. Numerical Implementation

The numerical implementation of the Direct FE^2^ method accounting for coupled thermo-mechanical effects is briefly reviewed below. The adopted procedure generally follows the established framework introduced in [28] and extended to thermo-mechanical problems by Zhi et al. [33,35]. Nevertheless, the present study adapts this framework to the specific case of heterogeneous steel partition walls, where the structural configuration and thermal boundary conditions require tailored treatments. The novelty of this work thus lies not in reformulating the FE^2^ method itself, but in extending and applying it to investigate the multi-scale thermo-mechanical responses of steel partition walls under elevated temperatures.

### 3.1. Boundary Conditions

To satisfy the kinematic boundary conditions for the displacement and temperature fields in Equation (6), PBCs need to be prescribed to mesoscale RVEs, and they can be derived from Equation (7) as follows:

(18) $${\hat{u}}_{1}{|}_{R}-{\hat{u}}_{1}{|}_{L}=2l_{1}\nabla_{1}u_{1}\;{\hat{u}}_{2}{|}_{R}-{\hat{u}}_{2}{|}_{L}=2l_{1}\nabla_{1}u_{2}\;\hat{T}{|}_{R}-\hat{T}{|}_{L}=2l_{1}\nabla_{1}T\;{\hat{u}}_{1}{|}_{T}-{\hat{u}}_{1}{|}_{B}=2l_{2}\nabla_{2}u_{1}\;{\hat{u}}_{2}{|}_{T}-{\hat{u}}_{2}{|}_{B}=2l_{2}\nabla_{2}u_{2}\;\hat{T}{|}_{T}-\hat{T}{|}_{B}=2l_{2}\nabla_{2}T$$

where the macro-scale displacement $u=[u_{1},u_{2}{]}^{T}$ and temperature T can be expressed using the nodal displacements $a_{u}$, nodal temperatures $a_{T}$ and their corresponding shape functions N as

(19) $$u=Na_{u}\;T=Na_{T}$$

To prevent rigid body translation of the RVE and temperature mismatch between the RVE and the macro-element, the displacements and temperature of the RVE’s central node are enforced to be equal to the macro-scale values at the corresponding Gaussian point of the macro-element, i.e.,:

(20) $${\hat{u}}_{i}{|}_{{\hat{x}}_{0}}=u_{i}\left(x_{GP}\right)\;\hat{T}{|}_{{\hat{x}}_{0}}=T\left(x_{GP}\right)$$

where ${\hat{x}}_{0}$ represents the coordinates of the central node of the RVE, and $x_{GP}$ represents the coordinates of the corresponding Gauss point of the macro-element.

Note that the constraints defined in Equations (18) and (20) can be directly implemented into the multiscale finite element model using MPCs, a common feature in many commercial software, such as ABAQUS. This significantly simplifies the numerical implementation of the Direct FE^2^ method and facilitates its application to large-scale partition wall simulations.

### 3.2. Enforcement of the Hill-Mandel Condition

For a coupled thermomechanical problem, variation in the total internal energy can be expressed as

(21) $$\delta H=\int_{V}\;\delta H_{\mathrm{den}}dV$$

or in the following discretized form:

(22) $$\delta H=\sum_{\alpha }\;w_{\alpha }J_{\alpha }(\delta H_{den}{)}_{\alpha }$$

where α represents the Gaussian integration point, and $J_{\alpha }$ and $w_{\alpha }$ represent the Jacobian and the Gaussian integration weight, respectively.

According to the Hill–Mandel condition (see Equation (15)) and the volume average of meso-scale internal energy (see Equation (5)), Equation (22) can be rewritten as

(23) $$\delta H=\sum_{\alpha }\;w_{\alpha }J_{\alpha }\langle \delta \hat{H}\rangle_{\alpha }=\sum_{\alpha }\;\frac{w_{\alpha }J_{\alpha }}{\left|{\hat{V}}_{\alpha }\right|}\int_{{\hat{V}}_{\alpha }}\left({\hat{\sigma }}_{ij}\delta {\hat{u}}_{i,j}+\hat{\rho }\dot{\hat{U}}\delta \hat{T}+{\hat{q}}_{i}\delta {\hat{T}}_{,i}\right)d{\hat{V}}_{\alpha }$$

Note that the Direct FE^2^ method prescribes zero material properties to macro-elements, implying that the total internal energy is contributed by only meso-scale RVEs, i.e.,

(24) $$\delta H=\sum_{\alpha }\;\delta {\hat{H}}_{\alpha }=\sum_{\alpha }\;\int_{{\hat{V}}_{\alpha }}\left({\hat{\sigma }}_{ij}\delta {\hat{u}}_{i,j}+\hat{\rho }\dot{\hat{U}}\delta \hat{T}+{\hat{q}}_{i}\delta {\hat{T}}_{,i}\right)d{\hat{V}}_{\alpha }$$

Comparison between Equations (23) and (24) indicate that

(25) $${\tilde{w}}_{\alpha }=\frac{w_{\alpha }J_{\alpha }}{\left|{\hat{V}}_{\alpha }\right|}=1$$

This implies that enforcement of the Hill–Mandel condition requires scaling of the RVE’s volume such that $\left|{\hat{V}}_{\alpha }\right|=w_{\alpha }J_{\alpha }$. For 2D problems, this can be performed easily by scaling the thickness of RVE, while for 3D problems, the RVE needs to be scaled equally in three dimensions.

It is worth noting that, while the numerical enforcement of periodic boundary conditions and the Hill–Mandel condition is consistent with previous FE^2^ studies, the present work adapts and extends this implementation to the specific case of steel partition walls with heterogeneous configurations. This tailored application ensures accurate thermo-mechanical coupling at multiple scales and demonstrates the applicability of the Direct FE^2^ framework to engineering-scale structural systems, which has not been explicitly addressed in earlier studies.

## 4. Applications for Partition Walls

The Direct FE^2^ method was then applied to simulate the coupled thermomechanical responses of three different partition walls, consisting of solid steel plates, hollow steel plates with voids, and fiber-reinforced composite plates. All case studies were conducted through secondary development in ABAQUS finite element software using Python 2.7, enabling the implementation of the Direct FE^2^ multiscale modeling framework.

### 4.1. Solid Steel Plate

**Figure 2:**
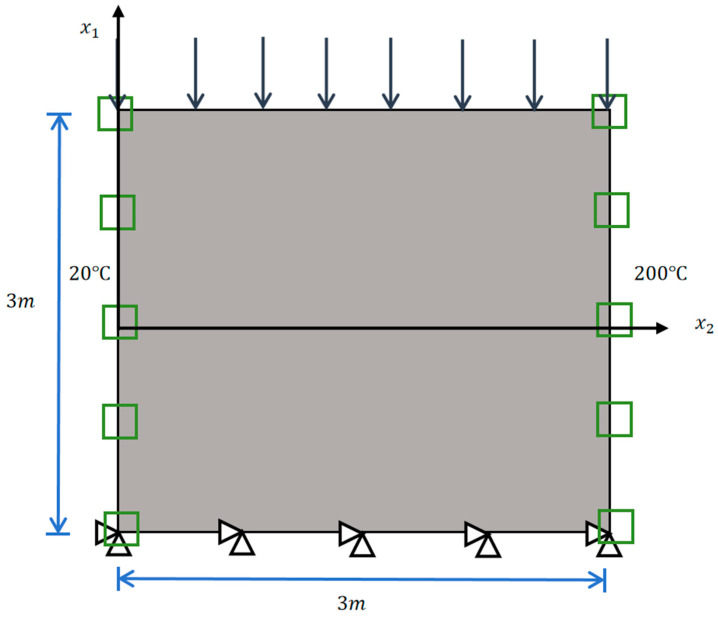
Illustration of a solid steel plate and its loading and boundary conditions.

To validate the accuracy of the Direct FE^2^ method in capturing coupled thermomechanical responses, a homogeneous steel plate (3 m × 3 m × 20 mm), commonly used as a partition wall, was first simulated. Constant temperatures of 20 °C and 200 °C were applied to the left and right edges, respectively. A uniform pressure load of 5 N/mm^2^ was applied to the top edge, while the bottom edge was fixed and thermally insulated (as shown in Figure 2). The material properties of the steel are provided in Table 1.

**Figure 3:**
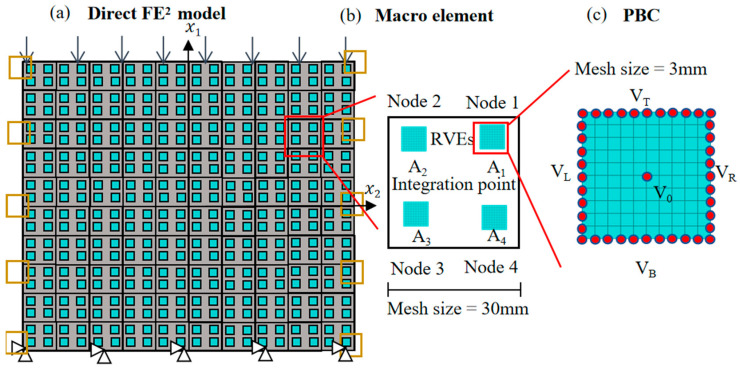
Schematic of the Direct FE^2^ modeling framework. (**a**) Macro model with embedded RVEs (blue squares). (**b**) Representative macro element with four integration points (A_1_–A_4_), each linked to an RVE. (**c**) Periodic boundary conditions (PBC) applied to an RVE.

In the Direct FE^2^ model, the solid steel plate was first meshed using coarse macro-elements with zero material properties to enforce Equation (24). RVEs (30 mm × 30 mm × 20 mm) containing the actual material properties were then embedded at the Gauss points of each macro-element, as illustrated in Figure 3. Periodic boundary conditions (PBCs) were applied to link the displacements of the four vertex nodes of each macro-element (Node1, Node2, Node3, and Node4 in Figure 3) with the corresponding boundary nodes of the RVEs (VT, VB, VL, and VR). Finally, boundary conditions were directly applied at the macroscale. As the Direct FE^2^ model requires only a limited number of RVEs, it offers significant potential for high computational efficiency.

**Figure 4:**
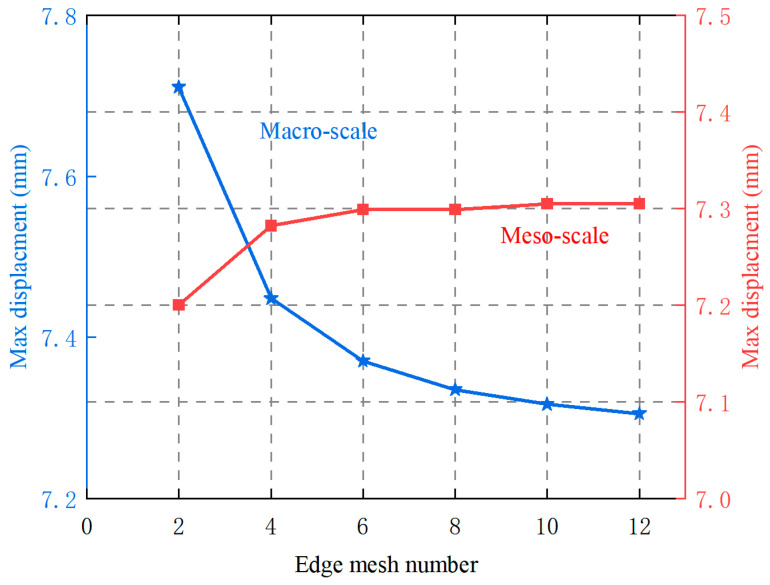
The effect of macro- and meso-scale mesh numbers on maximum displacement of top edge in Direct FE^2^ simulations.

A mesh sensitivity analysis was first conducted to minimize mesh dependence and determine appropriate mesh sizes. The Direct FE^2^ model involves two levels of discretization: macro-elements for the macroscopic geometry and meso-meshes for the RVEs. The macroscopic mesh sensitivity was examined first. Solid steel plates were meshed using CPS4T elements under the assumptions of steady-state heat conduction and negligible geometric nonlinearity. The number of nodes along each macroscopic edge ranged from 2 to 12 in increments of 2, while the number of nodes along each mesoscopic edge was fixed at 10. As shown in Figure 4, the maximum displacement of the top edge converged satisfactorily when the macroscopic mesh exceeded 10 nodes per edge. Therefore, a 10 × 10 macroscopic mesh was adopted.

For the RVEs, mesh sizes were varied from 2 × 2 to 12 × 12 elements. Figure 4 also shows that the maximum top-edge displacement converged when the RVE mesh exceeded 10 × 10 elements. Accordingly, the mesoscale model was meshed with 10 × 10 elements to ensure accuracy while reducing mesh sensitivity. For consistency, the DNS model used the same mesh size as the mesoscale mesh in the Direct FE^2^ model.

**Figure 5:**
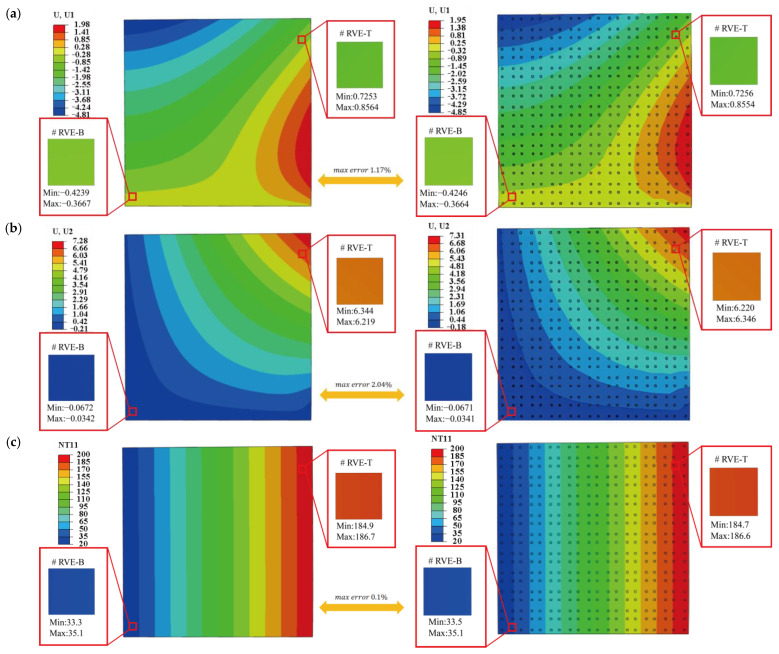
Comparison of displacement components and temperature obtained from DNS and Direct FE2 method (**a**) Displacement contours of the partition wall plate in the $x_{1}$-direction (**b**) Displacement contours of the partition wall plate in the $x_{2}$-direction, and (**c**) Temperature contours of the partition wall plate.

Figure 5a and Figure 5b present the displacement contours in the $x_{1}$ and $x_{2}$-directions, respectively, obtained from the DNS and Direct FE^2^ models. Specifically, the contour of U1 in Figure 5a shows a gradual increasing pattern in displacement field from left to right, with the maximum occurring near the high-temperature edge. This pattern reflects thermal expansion induced by the temperature difference between the left (20 °C) and right (200 °C) edges. As the right-side experiences greater thermal elongation, the plate expands horizontally toward the left. In contrast, Figure 5b illustrates the U2 displacement due to the vertical pressure load. The largest vertical displacement appears along the top edge, with a downward trend consistent with the direction of the applied load. The bottom edge remains fixed, resulting in a vertically symmetric bending deformation. The deformation directions and magnitudes are consistent between the two approaches and the relative difference between those two results are 1.17% and 2.04% for U1 and U2, confirming the accuracy of the Direct FE^2^ method in reproducing the overall structural behavior.

Figure 5c compares the temperature distributions from the two models. A smooth, nearly linear gradient from left to right aligns well with the applied thermal boundary conditions. The absence of localized temperature concentrations confirms steady-state heat conduction and homogeneous material behavior. The temperature fields from both models show excellent agreement, with a maximum relative difference of less than 0.1%, further validating the thermal coupling accuracy of the Direct FE^2^ approach. Overall, these results demonstrate that the Direct FE^2^ method effectively captures multiscale thermomechanical behavior with high accuracy and computational efficiency.

### 4.2. Circular Hole Steel Plate

**Figure 6:**
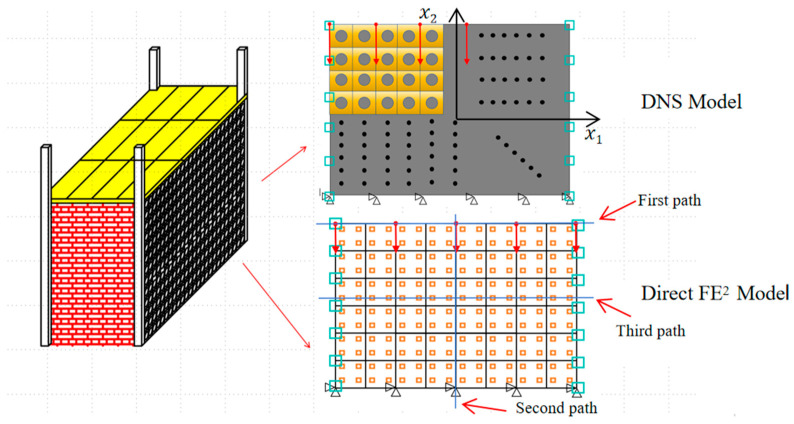
Schematic diagram of the DNS model and Direct FE^2^ model.

A more practical example, perforated steel plates often employed for reducing dead weight, was investigated in this section and it was shown in Figure 6. The engineering structure was simplified as a plate with a dimension of 4 m × 3 m × 20 mm where the hole with a diameter of 6.25 mm was uniformly distributed with a spacing of 50 mm in both two directions.

In practical applications, such structures are often subjected to significant temperature gradients, with one side exposed to high temperatures (~200 °C) and the opposite side maintained at a lower temperature (~20 °C). This leads to deformation due to thermomechanical coupling effects. To replicate this scenario, a vertical displacement of 20 mm was applied at the top edge of the partition wall, simulating the influence of a supporting beam. The bottom edge was fully constrained in all degrees of freedom, while the left and right edges were maintained at 20 °C and 200 °C, respectively. Both the top and bottom edges were assumed to be adiabatic.

Thermomechanical coupling analyses were carried out using both the Direct FE^2^ method and the DNS approach to examine the deformation characteristics and stress distribution within the structure. The material properties used in this example are consistent with those listed in Table 1 of the previous section. In addition, the plasticity parameters given in Table 2 were incorporated. Notably, the simulations accounted for the temperature dependence of the elastic modulus, as described by Equation (26), to capture the material’s softening behavior at elevated temperatures.

(26) $$E=\left\{\begin{array}{c} 210,000\;,\;20\;{}^{\circ}C\\ \;\;210,000\;,\;100\;{}^{\circ}C\\ \;189,000\;,\;200\;{}^{\circ}C \end{array}\right.$$

**Figure 7:**
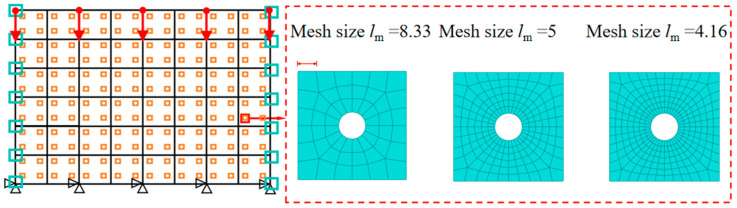
Schematic diagram of RVE with different mesh divisions.

**Figure 8:**
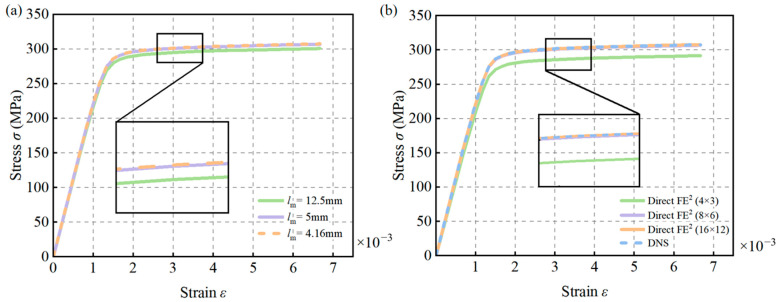
Influence of macro and mesoscale mesh sizes on structural stress–strain in Direct FE^2^ models: (**a**) Simulation results with different mesoscale element sizes; (**b**) Simulation results with different macroscale element sizes.

CPS4T elements in ABAQUS were used for both the macroscale and mesoscale models, and mesh sensitivity analyses were conducted for both scales. At the mesoscale, three different RVE sizes—8.33 mm, 5 mm, and 4.16 mm—were tested, as shown in Figure 7. The corresponding structural engineering stress–strain responses are presented in Figure 8a. The results indicate that when the element size is reduced below 5 mm, the differences in the predicted responses become negligible. Therefore, a mesoscale element size of 5 mm was selected for subsequent simulations.

For the macroscale model, different mesh number—4 × 3, 8 × 6, and 16 × 12 macro-elements—were considered. As shown in Figure 8b, the results reveal that the discrepancy between the DNS and Direct FE^2^ models becomes negligible once the macro mesh reaches 8 × 6 elements. Hence, this mesh configuration was adopted to achieve a balance between computational efficiency and accuracy in the following Direct FE^2^ analyses.

To analyze displacement and temperature distributions in the thermo-mechanical coupling model, results were extracted along three representative paths (see Figure 6). The first path, at the structure’s top boundary, examines displacement and thermal stress near the top region. The second, through the structure’s center (*x*_1_ = 0 mm), focuses on vertical deformation and related thermal stresses. The third, at *x*_2_ = 125 mm, crosses a row of RVEs to study local effects from material heterogeneity.

**Figure 9:**
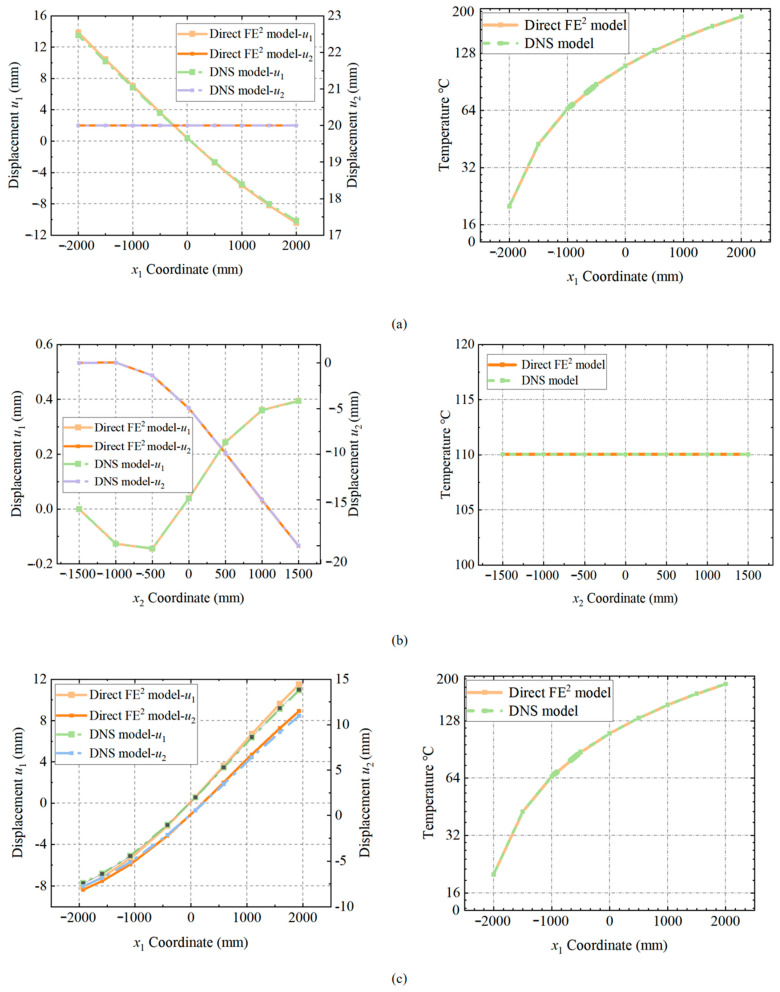
Comparison of displacement and temperature distributions between the Direct FE^2^ and DNS models along (**a**) the first path, (**b**) the second path, and (**c**) the third path.

Figure 9 compares displacement and temperature results from the Direct FE^2^ and DNS models along these paths. Along the first path (Figure 9a), vertical displacement *u*_2_ is constant due to the uniform vertical boundary condition, while horizontal displacement *u*_1_ initially decreases with increasing *x*_1_, reaching a minimum at *x*_1_ = 0, and then increases as *x*_1_ continues to grow. This pattern arises due to temperature-induced softening reducing local stiffness at first, and then the material’s behavior changes past the center of the model. Both models show a linear temperature rise from 20 °C to 200 °C, reflecting steady-state heat conduction and confirming that horizontal deformation is mainly due to thermal expansion.

On the second path (Figure 9b), vertical displacement *u*_2_ increases with *x*_2_ in both models, indicating deformation due to vertical compression. Horizontal displacement *u*_1_ initially increases and then decreases, with the displacement continuing to increase in the opposite direction after *x*_2_ = 0. This suggests that the temperature-induced softening effect changes near *x*_2_ = 0, causing a reversal in the direction of horizontal displacement. The temperature along this path remains nearly constant at approximately 140 °C, indicating thermal equilibrium in the central region, with minimal influence of temperature gradients on vertical deformation

Along the third path (Figure 9c), both models show that *u*_1_ and *u*_2_ decrease with the *x*_1_ coordinate. At approximately *x*_1_ = 0, both *u*_1_ and *u*_2_ directions change, after which they continue to increase. The DNS model captures more pronounced local variations in *u*_2_ near the center, reflecting stress concentrations around perforations, whereas Direct FE^2^ provides a smoother but still accurate overall deformation. The temperature profile here is also uniform, reinforcing those lateral thermal gradients largely control the structural response.

**Figure 10:**
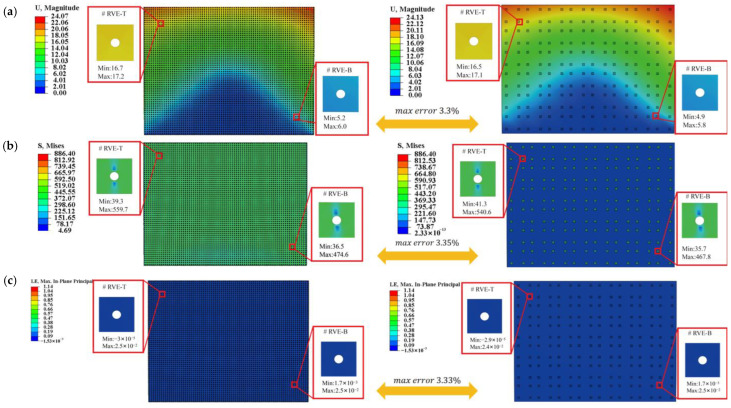

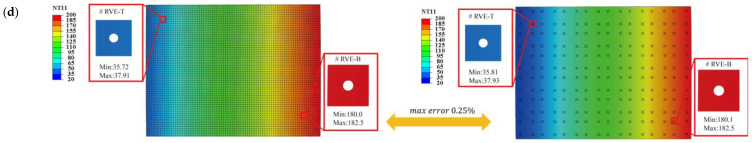
Displacement, stress and temperature contours at points E (−1875, 1375) and F (1875, −1375) for the selected RVE with compression displacement $u_{c}=20\;mm$; (**a**) Displacement obtained from DNS and Direct FE^2^; (**b**) Mises stress obtained from DNS and Direct FE^2^; (**c**) Max in plant principal obtained from DNS and Direct FE^2^. (**d**) Temperature obtained from DNS and Direct FE^2^.

For microscale validation, displacement, stress, max in plant principal, and temperature distributions within the RVE were compared (Figure 10) at two macroscopic points near the steel plate’s corners. The Direct FE^2^ and DNS results agree closely, with maximum differences below 3.04%. Both models capture localized stress concentrations around RVE voids and smooth temperature gradients, with slight distortions near void boundaries. These findings highlight that stress concentrations around perforations can be critical sites for damage or fatigue. The Direct FE^2^ method effectively captures these multi-scale effects, providing a reliable and efficient tool for evaluating the safety of perforated or thermally exposed structures.

### 4.3. Fiber-Reinforced Composite Plate

**Figure 11:**
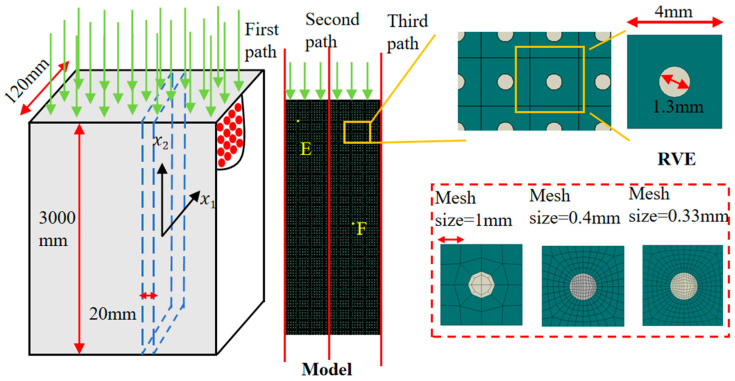
Schematic diagram of boron fiber reinforced aluminum plate and RVE mesh division.

Boron fiber-reinforced aluminum matrix (B/Al) composites are increasingly used in building partition systems due to their high performance and multifunctionality. In this study, a partition wall model with a dimension of 3000 mm × 120 mm × 20 mm (see Figure 11) is analyzed using a two-dimensional plane strain approach. Each representative volume element (RVE) has dimensions of 4 mm × 4 mm × 20 mm, with a centrally embedded circular fiber of 1.3 mm diameter. To ensure energy consistency, the thickness is scaled to 281 mm. For boundary conditions, the wall’s left edge is kept at 20 °C and the right edge at 200 °C, with the top and bottom edges adiabatic. A vertical displacement of 20 mm is applied to the top surface, while the bottom edge is fully constrained. The material properties for the boron fiber and aluminum matrix are listed in Table 3

**Figure 12:**
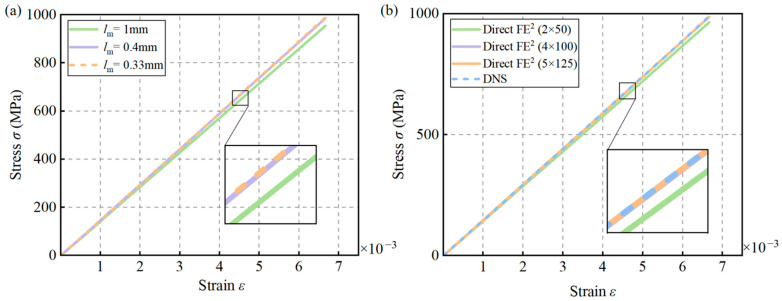
Influence of macro and mesoscale mesh sizes on overall structural stress–strain in Direct FE^2^ models: (**a**) Simulation results with different mesoscale element sizes; (**b**) Simulation results with different macroscale element numbers.

The RVEs were meshed using 4 × 100 CPE4T elements, with mesoscale mesh sizes of 1 mm, 0.4 mm, and 0.33 mm tested. As shown in Figure 12a, reducing the mesh size below 0.4 mm had negligible impact on the stress–strain curve. Therefore, a mesh size of 0.4 mm was selected for both the Direct FE^2^ and DNS models.

A mesh sensitivity analysis was also performed at the macroscale using 2 × 50, 4 × 100, and 6 × 150 elements. Figure 12b shows that increasing the number of macro-elements to 4 × 100 resulted in minimal differences in stress–strain responses between the DNS and Direct FE^2^ models. Thus, 4 × 100 macro-elements were used in subsequent simulations to ensure computational efficiency without sacrificing accuracy.

**Figure 13:**
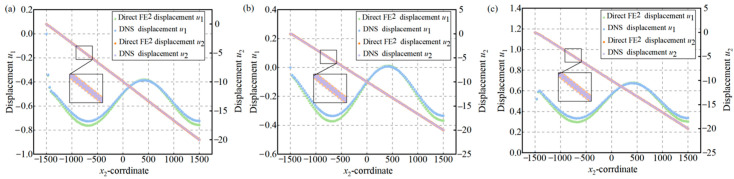
(**a**) Displacement distributions along the left edge; (**b**) Displacement distributions along the middle edge. (**c**) Displacement distributions along the right edge.

Figure 13 compares the displacement fields from Direct FE^2^ and DNS along three vertical paths in the composite partition wall: the left boundary, center, and right boundary. Displacement values are plotted against the *x*_2_-coordinate to directly assess the agreement between the two methods across the structure.

For all three paths, the vertical displacement *u*_2_ shows an approximately linear distribution, reflecting the applied compressive displacement at the top. In contrast, the horizontal displacement *u*_1_ displays a sinusoidal pattern due to stress redistribution and periodic buckling triggered by material heterogeneity. The Direct FE^2^ method accurately captures both the overall deformation and the local displacement oscillations, showing excellent agreement with the DNS results. Notably, the wave-like variation in *u*_1_ caused by the periodic fiber distribution is well reproduced by the multiscale model.

**Figure 14:**
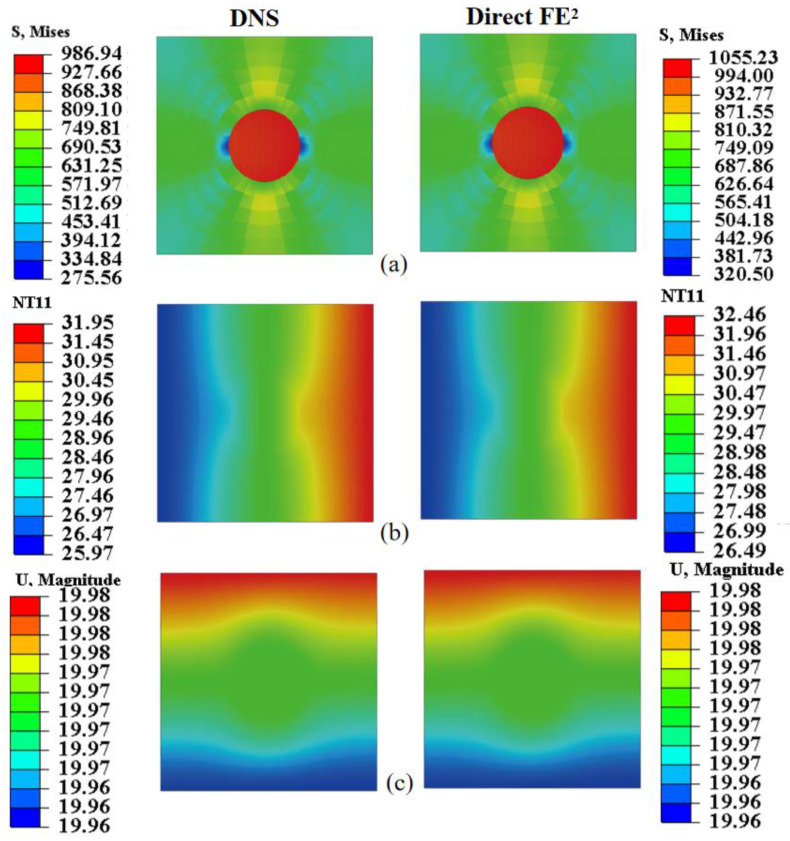
Local comparison of (**a**) von Mises stress, (**b**) temperature, and (**c**) displacement at RVE located at (−52, 1496) between DNS and Direct FE^2^ simulations.

**Figure 15:**
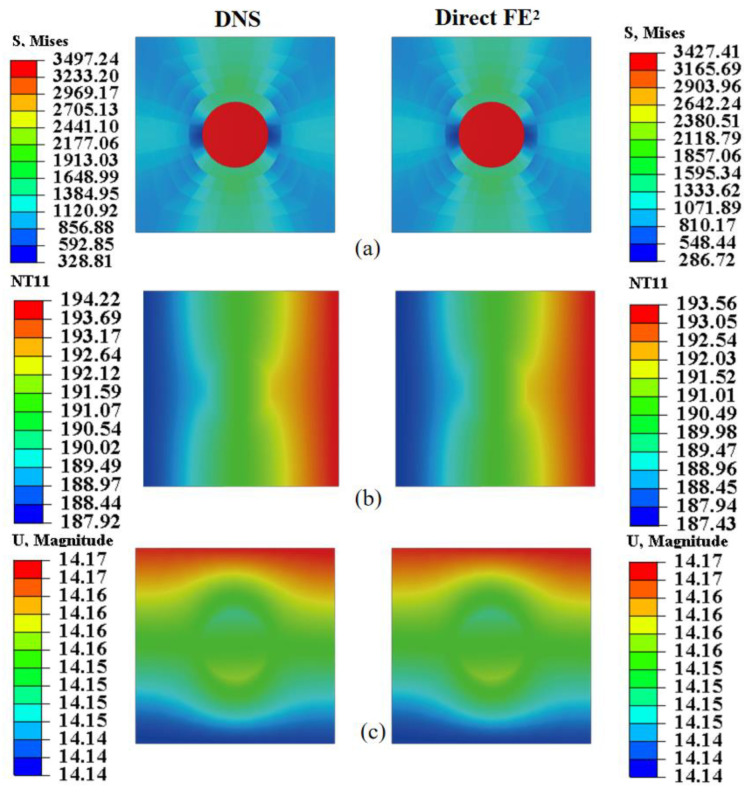
Local comparison of (**a**) von Mises stress, (**b**) temperature, and (**c**) displacement at RVE located at (56, 628) between DNS and Direct FE^2^ simulations.

Figure 14 and Figure 15 assess the local accuracy of the Direct FE^2^ method by comparing stress, temperature, and displacement fields within RVEs at points E (−52, 1496) and F (56, 628), each located in regions with different thermal and mechanical gradients. In both cases, Direct FE^2^ predictions closely match DNS results, with maximum relative errors under 5%.

Within each RVE, significant stress differences are observed between the fiber and matrix phases, mainly due to the large mismatch in elastic modulus and thermal expansion. The stiffer boron fiber bears higher stress, while the more compliant matrix undergoes greater deformation, causing interfacial stress discontinuities. These microscale stress variations, resulting from load transfer and thermal mismatch, are accurately captured by the multiscale model. This level of accuracy is crucial for reliable damage prediction and structural assessment of fiber-reinforced composites under coupled thermomechanical loading.

The Direct FE^2^ approach enables efficient multiscale simulation, making it a reliable tool for designing thermally loaded composite walls and structural components where local field variations can impact overall performance.

### 4.4. Transient Thermo-Mechanical Coupling Analysis

**Figure 16:**
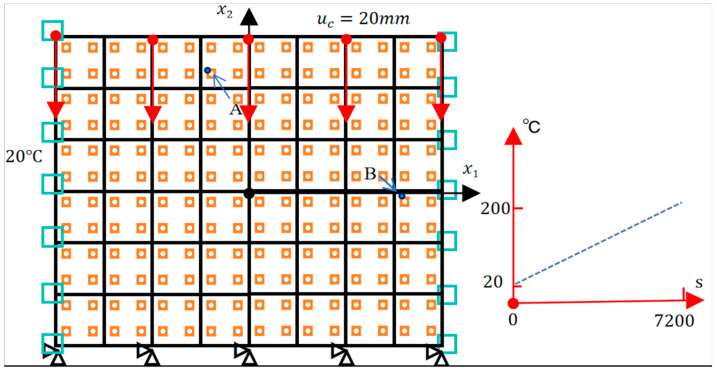
Schematic Diagram of Model Boundary Conditions.

As discussed in Section 2, the proposed Direct FE^2^ framework leverages the transient analysis solver in ABAQUS to accurately simulate the time-dependent response of coupled thermo-mechanical problems. To evaluate its performance under transient thermal loading, the model from Section 4.2 is extended for a transient analysis. The right boundary temperature increases linearly from 20 °C to 200 °C over the first 7200 s, then remains constant up to 86,400 s. The left boundary is held at 20 °C, while the top and bottom boundaries are adiabatic. A vertical displacement of 20 mm is applied to the top boundary to represent mechanical loading. Temperature-dependent material properties (see Table 4) are used to realistically capture thermal-mechanical coupling. All other conditions follow the original quasi-static setup shown in Figure 16.

**Figure 17:**
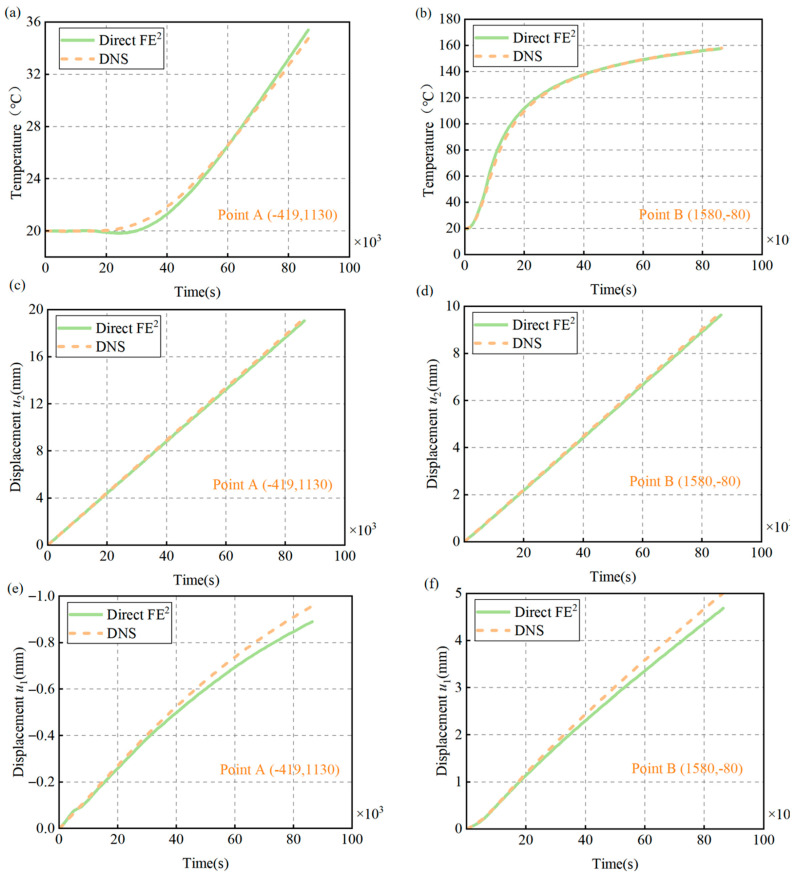
Comparison of displacement and temperature evolution at Points A (–419,1130) and B (1580, –80) under transient thermal loading, obtained from DNS and Direct FE^2^ models. (**a**) Temperature evolution at Point A; (**b**) Temperature evolution at Point B; (**c**) Vertical displacement $u_{2}$ at Point A; (**d**) Vertical displacement $u_{2}$ at Point B; (**e**) Horizontal displacement $u_{1}$ at Point A; (**f**) Horizontal displacement $u_{1}$ at Point B.

Figure 17a–f show the transient evolution of temperature and displacement at two points (A and B) in the structure under coupled thermo-mechanical loading. Point A (–419, 1130) is near the cold boundary, while Point B (1580, –80) is near the hot boundary, representing regions with different thermal environments.

Figure 17a,b display the temperature histories at these points. At Point A, temperature rises gradually from 20 °C to about 35 °C, slowing as equilibrium is reached, in line with Fourier’s law. At Point B, next to the heated boundary, temperature quickly increases to 160 °C. The Direct FE^2^ method accurately tracks these temperature changes, closely matching DNS results.

Figure 17c,d illustrate the vertical displacement (*u*_2_) evolution. Both points show a near-linear increase over time, reflecting steady thermal expansion. Point B experiences a faster increase due to higher temperatures, while Point A has smaller but proportional expansion. This behavior indicates that thermal strain dominates, consistent with the mechanical boundary conditions.

Figure 17e,f present the horizontal displacement (*u*_1_) at Points A and B from Direct FE^2^. At Point A, negative displacement occurs as the structure contracts near the cold side. At Point B, positive displacement results from local thermal expansion. Direct FE^2^ closely matches DNS in early and middle stages, but slightly underestimates displacement later on. The possible reason for this phenomenon is the Direct FE^2^ method cannot precisely capture the mechanical deformation induced by the thermal stress concentrated.

Overall, the favorable agreement between Direct FE^2^ and DNS across all responses demonstrates the effectiveness of the multiscale framework in capturing transient thermo-mechanical behavior. The method accurately resolves both global temperature evolution and local deformation. Further improvements, such as a finer macro-mesh or higher-order elements, could enhance the accuracy [43], but the results confirm the robustness and accuracy of the Direct FE^2^ approach for complex transient problems.

## 5. Computational Efficiency of the Direct FE^2^ Method

This chapter aims to systematically evaluate the computational efficiency of the Direct FE^2^ method in multiscale thermo-mechanical coupling analysis. Through comparative numerical examples, its advantages are demonstrated in terms of the number of elements, degrees of freedom, and computational time. Furthermore, by analyzing the time distribution across pre-processing, computation, and post-processing stages, the overall efficiency and practicality of the method in engineering applications are comprehensively highlighted.

First, the computational scale and total computational time of the four numerical examples in this study were statistically compared between the direct finite element simulations and the DNS method (see Table 4). Case 1 concerns the steady thermo-mechanical analysis of a solid steel plate presented in Section 4.1; Case 2 addresses the steady thermo-mechanical analysis of a steel plate with a circular hole; Case 3 focuses on the steady thermo-mechanical response of a fiber-reinforced composite plate; and Case 4 investigates the transient thermo-mechanical coupling behavior of a steel plate with a circular hole. All simulations were performed under identical hardware conditions, using a system equipped with 20 Intel Core i9-10900X processors (Intel, Santa Clara, CA, USA) (3.70 GHz) and 64 GB of RAM, with three CPU cores assigned to each simulation task. The total computational time was extracted from the log files recorded by the ABAQUS job manager. It should be emphasized that the commercial ABAQUS software required only a limited number of tokens to perform the analysis on a standard workstation. In contrast to the relatively low computational cost of the Direct FE^2^ model, the DNS model not only demands a significantly larger number of tokens to solve the same problems but also requires high-performance computing platforms for large-scale engineering applications, leading to substantially higher computational costs.

The results in Table 5 demonstrate that the Direct FE^2^ model exhibits a pronounced advantage over the DNS model in terms of computational scale. Specifically, its number of elements and degrees of freedom account for only a very small fraction of those in DNS, typically less than 5%. This difference indicates that, while ensuring computational accuracy, the Direct FE^2^ method can substantially reduce the discretization burden of the model, thereby alleviating both the storage demand and computational pressure of the solver. At the same time, the computational time of the Direct FE^2^ model is also markedly lower than that of DNS. Under identical hardware conditions, DNS requires handling a vast number of degrees of freedom and highly refined meshes, leading to a steep increase in computational cost. In contrast, Direct FE^2^ replaces full-scale mesh refinement with RVEs embedded at macroscopic integration points, thereby avoiding high-density global discretization and fundamentally reducing the number of iterations and overall solution cost. Consequently, this method consistently delivers orders-of-magnitude improvements in efficiency across the numerical examples, with its advantages becoming even more prominent in the analysis of complex structures. 

**Figure 18:**
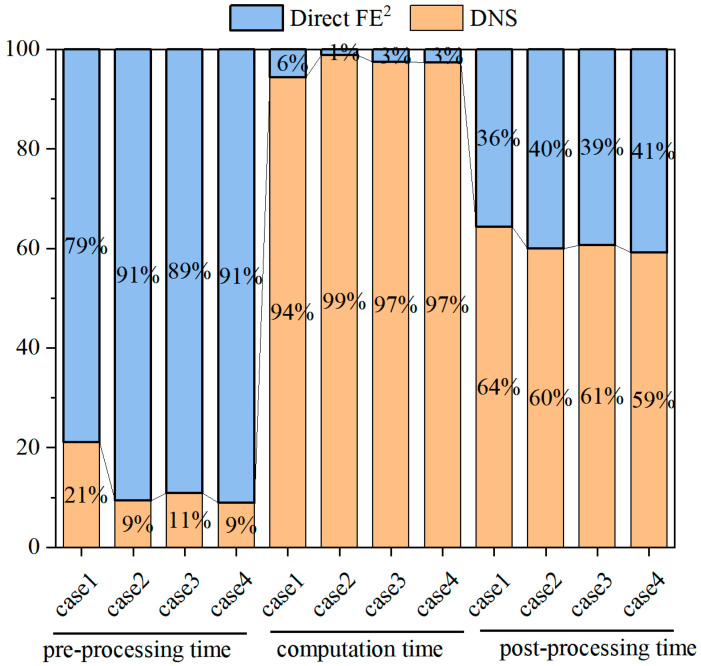
Comparison of computational time between Direct FE^2^ and DNS across the four numerical examples.

To more clearly illustrate the time consumption at each stage, this study further decomposed the process into pre-processing, computation, and post-processing, and they were compared in Figure 18 in percentage format (e.g., $t_{DNS}=T_{DNS}/\left(T_{DNS+DFE2}\right)$, where *t* and *T* represent the relative time and real time, respectively) to improve visibility due to large differences in their absolute values [44,45]. For comparison of post-processing efficiency, the time was defined as the duration required to extract the stress at a single node. As shown in the figure, the Direct FE^2^ method requires slightly more time during the pre-processing stage than DNS, owing to the additional tasks of constructing representative volume elements (RVEs), defining material parameters, and applying periodic boundary conditions. Nevertheless, this minor drawback is more than compensated for during the subsequent computation stage. By embedding RVEs at macroscopic integration points instead of discretizing the entire structure at a fine scale, Direct FE^2^ significantly reduces the number of elements and degrees of freedom, thereby drastically shortening the overall computational time. By contrast, DNS necessitates fine-scale meshing of the entire structure and explicit resolution of microstructural features, resulting in a heavy computational burden and a dramatic increase in processing time.

Compared with the classical DNS method, the main additional effort in the Direct FE^2^ model lies in establishing the kinematic relationship between the macro- and micro-models, which can be efficiently implemented through a predefined Python script. Apart from this step, the procedures for geometry modeling, mesh sensitivity analysis, and boundary condition specification remain largely similar to those in the DNS approach.

Overall, the Direct FE^2^ method demonstrates a significant advantage not only in terms of total computational time but also in the convenience of both pre- and post-processing. In particular, once a standardized scripting framework is established, the construction of RVEs and the application of boundary conditions can be highly automated, thereby further enhancing modeling efficiency and ensuring the reproducibility of results. Hence, the method exhibits remarkable potential for application to large-scale multiscale thermo-mechanical coupling problems.

## 6. Discussion of Engineering Implications and Limitations

The numerical results presented in this study provide insights into the multiscale thermomechanical responses of solid steel, perforated steel, and fiber-reinforced composite partition walls. From an engineering perspective, these findings highlight that the Direct FE^2^ method enables more efficient fire-resistance evaluation and structural safety assessment than conventional DNS approaches, particularly for large-scale wall systems used in modern buildings and industrial facilities. The method also offers practical advantages in supporting the design of lightweight and energy-efficient wall systems, where perforations and composite materials are increasingly adopted to achieve superior thermal and mechanical performance.

Nevertheless, several limitations must be acknowledged. First, the present analysis assumes linear elastic material behavior and does not account for plasticity, creep, or damage evolution, which may become significant under severe thermal loading. Second, the boundary conditions adopted in the simulations are simplified compared with realistic fire scenarios, where non-uniform heating, transient flames, and load redistribution can strongly affect structural responses. Third, the current implementation neglects uncertainties in material properties and loading, which may lead to variations in predicted responses. These limitations suggest that further work is needed to extend the Direct FE^2^ framework toward nonlinear, time-dependent, and stochastic analyses in order to enhance its reliability for practical engineering design.

Overall, while the Direct FE^2^ method demonstrates promising potential for engineering applications, careful consideration of its assumptions and limitations is necessary when extrapolating the present results to real-world scenarios.

## 7. Conclusions

This study extended an efficient Direct FE^2^ method for addressing thermomechanical coupling in multiscale partition wall structures. Three representative cases—homogeneous, porous, and composite walls—were analyzed using the Direct FE^2^ approach, with DNS results employed as reference solutions. The key findings can be summarized as follows:

(a) A practical framework was established for static and transient thermomechanical analysis within common commercial finite element software.
(b) The Direct FE^2^ method showed less than 5% deviation from DNS results in both displacement and temperature, while achieving a computational efficiency improvement of approximately one order of magnitude.
(c) The simplified preprocessing requirements and high efficiency highlight its suitability for a wide range of engineering applications.

The proposed Direct FE^2^ method demonstrates strong potential for engineering practice due to its unique advantages:

(a) Evaluation and design of partition walls in high-rise buildings and industrial facilities.
(b) Optimization of lightweight and perforated wall systems through accurate prediction of thermal stress redistribution and local failure.
(c) Performance-based evaluation of fiber-reinforced composite walls under thermal loading, supporting the development of advanced structural materials.

In conclusion, the Direct FE^2^ method provides a reliable, efficient, and versatile strategy for multiscale thermomechanical analyses, offering a promising framework with broad applicability for both engineering practice and scientific research in computational mechanics.

## Figures and Tables

**Table 1:** Material Properties of Q335 Steel [41].

Parameters	Units	Value
Density ρ	$kg∕m^{3}$	7850
Elasticity modulus E	GPa	210
Poisson’s ratio v	−	0.3
Conductivity λ	w/(m·K)	45
Thermal expansion α	1/°C	$1.2\times 10^{-5}$

**Table 2:** Plasticity Parameters of Q335 Steel [41].

Yield Stress (σ)	Plastic Strain
362	0.00000
373	0.04702
383	0.07514
394	0.10248
401	0.12031
408	0.13782

**Table 3:** Material properties of boron fiber reinforced aluminum matrix (B/Al) composites [42].

Parameters	Units	Boron Fiber	Aluminum Matrix
Density ρ	$kg∕m^{3}$	2600	2700
Elasticity modulus E	GPa	385	75
Poisson’s ratio v	−	0.2	0.33
Conductivity λ	w/(m·K)	38	247
Thermal expansion α	1/°C	$5\times 10^{-6}$	$2.36\times 10^{-5}$

**Table 4:** Material Properties of Q335 Steel as Functions of Temperature [41].

Parameters	Units	Value	Temperature
Density ρ	$kg∕m^{3}$	7850	−
Elasticity modulus E	GPa	210	20 °C
		210	100 °C
		189	200 °C
Poisson’s ratio v	−	0.3	−
Conductivity λ	w/(m·K)	45	20 °C
		53	200 °C
Thermal expansion α	1/°C	1.2 × 10^−5^	20 °C
		1.264 × 10^−5^	100 °C
		1.344 × 10^−5^	200 °C
$\mathrm{Specific}\;\mathrm{heat}\;\mathrm{capacity}\;c_{p}$	J/(kg·°C)	440	20 °C
		690	200 °C

**Table 5:** Comparison of Computational Scale and Total Computational Time between Direct FE^2^ and DNS.

Numerical Case	Number of Elements		Degrees of Freedom		Total Computational Time (s)	
	Direct FE^2^	DNS	Direct FE^2^	DNS	Direct FE^2^	DNS
Case 1	40,000	1,000,000	120,000	3,630,000	85	530
Case 2	37,632	940,800	133,632	3,340,800	333	21,419
Case 3	409,600	5,760,000	1,329,600	18,697,500	706	3015
Case 4	37,632	940,800	133,632	3,340,800	296	10,700

## Data Availability

The original contributions presented in this study are included in the article. Further inquiries can be directed to the corresponding authors.
